# Copeptin, a novel prognostic biomarker in ventilator-associated pneumonia

**DOI:** 10.1186/cc6780

**Published:** 2008-02-02

**Authors:** Renato Seligman, Jana Papassotiriou, Nils G Morgenthaler, Michael Meisner, Paulo JZ Teixeira

**Affiliations:** 1 Hospital de Clínicas de Porto Alegre, Rua Ramiro Barcelos 2350, 90035-003 Porto Alegre, Brazil; 2 Universidade Federal do Rio Grande do Sul, Rua Ramiro Barcelos 2400 – 4o Andar, 90035-003 Porto Alegre, Brazil; 3 Research Department, BRAHMS AG, Neuendorfstrasse 25, D-16761 Hennigsdorf bei Berlin, Germany; 4 Hospital of Dresden–Neustadt, Industriestrasse 40, D-01129 Dresden, Germany

## Abstract

**Background:**

The present study sought to investigate the correlation of copeptin with the severity of septic status in patients with ventilator-associated pneumonia (VAP), and to analyze the usefulness of copeptin as a predictor of mortality in VAP.

**Methods:**

The prospective observational cohort study was conducted in a teaching hospital. The subjects were 71 patients consecutively admitted to the intensive care unit from October 2003 to August 2005 who developed VAP. Copeptin levels were determined on day 0 and day 4 of VAP. Patients were followed for 28 days after the diagnosis, when they were considered survivors. Patients who died before day 28 were classified as nonsurvivors. There were no interventions.

**Results:**

Copeptin levels increased from sepsis to severe sepsis and septic shock both on day 0 and day 4 (*P *= 0.001 and *P *= 0.009, respectively). Variables included in the univariable logistic regression analysis for mortality were age, gender, Acute Physiology and Chronic Health Evaluation II score and ln copeptin on day 0 and day 4. Mortality was directly related to ln copeptin levels on day 0 and day 4, with odds ratios of 2.32 (95% confidence interval, 1.25 to 4.29) and 2.31 (95% confidence interval, 1.25 to 4.25), respectively. In a multivariable logistic regression model for mortality, only ln copeptin on day 0 with odds ratio 1.97 (95% confidence interval, 1.06 to 3.69) and ln copeptin on day 4 with odds ratio 2.39 (95% confidence interval, 1.24 to 4.62) remained significant.

**Conclusion:**

Our data demonstrate that copeptin levels increase progressively with the severity of sepsis and are independent predictors of mortality in VAP.

## Introduction

Arginine vasopressin (AVP), produced by hypothalamic neurons, is stored and released from the posterior pituitary gland following different stimuli such as hypotension, hypoxia, hyperosmolarity, acidosis and infections [1]. AVP has vasoconstrictor and antidiuretic properties and has potency to restore vascular tone in vasodilatory hypotension [2]. AVP is derived from a larger precursor (preproAVP) along with two other peptides of unknown function, neurophysin II and copeptin, the carboxy-terminal part of the precursor [3]. Measurement of AVP levels has limitations due to its short half-life and instability. Copeptin is a more stable peptide. Copeptin concentrations mirror that of AVP and are also elevated in sepsis and septic shock [4]. In critically ill patients, copeptin values increased significantly with the severity of the disease [4-6]. The role of copeptin is as yet unclear. Copeptin was recently suggested to play an important role in the correct structural formation of the AVP precursor, as a prerequisite for its efficient proteolytic maturation [7].

In septic patients, copeptin was higher on admission in nonsurvivors as compared with survivors, suggesting copeptin may be a prognostic marker in sepsis [5].

Stolz and collaborators assessed the prognostic value of copeptin in acute exacerbation of chronic obstructive pulmonary disease [8]. Copeptin was predictive for long-term clinical failure independent of age, comorbidity, hypoxemia, and lung functional impairment. In that study copeptin was a prognostic marker for short-term and long-term prognosis in patients with acute exacerbation of chronic obstructive pulmonary disease requiring hospitalization [8].

Muller and collaborators studied copeptin in community-acquired pneumonia patients. Copeptin levels increased with increasing severity of community-acquired pneumonia. In patients who died, the copeptin levels on admission were significantly higher compared with levels in survivors [6].

No published information exists to date about the behavior of copeptin in patients with ventilator-associated pneumonia (VAP). The present study aimed to investigate the correlation of copeptin with the severity of septic status in patients with VAP, and to analyze the usefulness of copeptin as a predictor of mortality in VAP.

## Materials and methods

The study was conducted in the clinical/surgical 26-bed intensive care unit (ICU) of the Hospital de Clínicas de Porto Alegre, a tertiary-care–teaching institution with 744 hospital beds.

All patients consecutively admitted to the ICU suspected of VAP were eligible for this prospective observational cohort study. Patients at least 18 years old were recruited. The exclusion criteria were a previous diagnosis of AIDS or neutropenia <500 cells/ml. Pneumonia was considered ventilator-associated when it occurred after 48 hours of mechanical ventilation and was judged to not have been incubating before starting mechanical ventilation. VAP was considered early-onset when it occurred during the first 4 days of mechanical ventilation and was considered late-onset when it developed 5 days or more after the initiation of mechanical ventilation [9]. The Acute Physiology and Chronic Health Evaluation (APACHE) II score was calculated during the first 24 hours of admission to the ICU [10]. Patients were considered immunosuppressed when they had received chemotherapy within the preceding 45 days, or had neutropenia less than 1,000/mm^3^.

Diagnosis of pneumonia was suspected when a patient developed a new and persistent radiographic infiltrate plus two of the following signs/symptoms: body temperature >38°C or <36°C; white blood cells >11,000 or <4,000/mm^3^; and macroscopically purulent tracheal aspirate [11]. Purulent endotracheal aspirate was defined on inspection by the assistant team. The axillary temperature used was the highest in the previous 24 hours before inclusion into the study.

A chest X-ray scan, arterial blood gases, complete blood count, creatinine, total bilirubin, and albumin were obtained by the time VAP was suspected (D0) and were repeated on the fourth day of treatment (D4). Quantitative endotracheal aspirate (QEA) was obtained on D0, repeated on the third day after the diagnosis (D3) and then obtained weekly. Sterile endotracheal aspirates were obtained with a suction catheter adapted to a mucus collector without saline instillation, and two samples of hemocultures were collected from different veins with a 15-minute interval before starting antimicrobial treatment.

The Clinical Pulmonary Infection Score (CPIS) [12], modified as described by Singh and colleagues [13], was calculated on the basis of data on D0 and D3. Patients were assumed to have VAP when the CPIS was 7 points or more. The CPIS was calculated with data from D0, adding points for microbiological results and progression of pulmonary infiltrate on a new chest X-ray scan on D3. To calculate the CPIS on D3, data from D3 were used.

For a diagnosis of VAP there should be no evidence of another medical condition to which the presenting symptoms, signs or radiological findings could be attributed. A Sequential Organ Failure Assessment score was calculated on D0 and D4. QEA was considered positive when values were at least 10^5 ^colony-forming units/ml.

All patients with a clinical suspicion of VAP, later confirmed by a CPIS of at least 7 points and fulfilling inclusion criteria, were included and received empirical antimicrobial therapy on D0. The choice of antibiotics and changes rested solely with the critical care team or primary service caring for the patient. Modifications to empirical therapy were based on the results of QEA and hemocultures. Mechanical ventilation, physiotherapy and airway management were performed in accordance with a standard protocol in all patients.

Patients were classified at the time of VAP diagnosis into those with sepsis, those with severe sepsis and those with septic shock, which were defined according to international criteria [14,15].

Patients' progress was followed until the 28th day (D28) after the diagnosis of VAP. Patients who survived until follow up were counted as survivors. Assuming crude mortality, patients who died before D28 were nonsurvivors. Patients discharged from the ICU before D28 were also considered survivors. All patients with VAP were reviewed by one of the investigators to confirm the diagnosis on the basis of predetermined criteria.

Seventy-one patients enrolled from October 2003 to August 2005 constituted the study population. The research protocol was reviewed and approved by the Human Research Committee from the Hospital de Clínicas de Porto Alegre, and informed written consent was obtained from patients' representatives before enrollment. The study protocol conforms to the ethical guidelines of the Declaration of Helsinki.

Trained investigators collected data on D0, on D3, on D4, and weekly until D28. The recorded data included age, sex, cause of ICU admission, arterial partial pressure of oxygen/fraction of inspired oxygen, APACHE II score, Sequential Organ Failure Assessment score, CPIS, comorbidities including chronic obstructive pulmonary disease, whether an active smoker, history of congestive heart failure, history of malignancy, immunosuppression, albumin, use of histamine type-2 receptor antagonist, use of proton pump inhibitor, use of corticosteroids, dialysis, central vein catheterization, urinary tract catheterization, duration of mechanical ventilation, duration of stay in ICU before VAP, cardiopulmonary resuscitation, intubation (orotracheal versus nasotracheal), and tracheotomy.

Adequacy of the empirical antimicrobial treatment was recorded on the basis of microbiological results. Adequate antibiotic therapy was defined as coverage of all the pathogens isolated (from the QEA culture or from blood), by at least one antimicrobial administered by the onset of VAP, determined by the sensitivity pattern in the antibiogram [16]. Treatment was considered adequate when cultures were negative.

Blood was drawn when a diagnosis of VAP was clinically suspected, before empirical antibiotic treatment was started. Samples of serum were prepared and frozen immediately after blood was drawn, and then stored at -80°C in the Hospital de Clínicas de Porto Alegre research laboratory. Assays were performed in batches at the end of the study period.

Copeptin measurements were performed in D0 and D4 samples using a new sandwich immunoluminometric assay, as described recently [17]. Briefly, two polyclonal antibodies to the C-terminal region (covering amino acids 132 to 164 of pre-proAVP) were used. One antibody is bound to polystyrene tubes, and the other is labeled with acridinium ester for chemiluminescence detection. The assay requires 50 μl serum or plasma and yields results within 3 hours. In contrast to measurements of mature AVP, no extraction step prior to measurement is needed and the analyte shows *ex vivo *stability for at least 7 days at room temperature and for 14 days at 4°C. The assay has a functional assay sensitivity (defined as the lowest value with an interassay coefficient of variation <20%) of 2.25 pmol/l. The median copeptin level in 359 healthy individuals in previous investigations was 4.2 pmol/l [17].

Copeptin measurements were performed in the Research Department of BRAHMS AG (Biotechnology Centre, Hennigsdorf/Berlin, Germany). Laboratory measurements were performed in a blinded fashion without knowledge of the clinical status of the patient.

### Statistical analysis

Continuous baseline data are expressed as the means ± standard deviation. Categorical variables were compared with the chi-squared test. Comparison of the copeptin levels between survivors and nonsurvivors was analyzed by the Mann–Whitney test. Comparison of the copeptin levels in different septic status patients was analyzed by the Kruskal–Wallis test. For these analyses, two-tailed tests and *P *≤ 0.05 were considered statistically significant.

Logistic regression analysis was used to determine the relation of risk factors to clinical outcome. We performed logarithmic transformation of copeptin values in the regression models, since they have a nonparametric distribution. In a multivariable model we considered significant variables with biological importance. Variables with *P *< 0.20 in univariable logistic regression were entered into the multivariable model. In the multivariable model we considered as significant those variables with *P *< 0.05.

SPSS 11.0 for Windows (SPSS Inc., Chicago, IL, USA) was used for statistical analysis.

## Results

Seventy-one patients were included in the study. Forty-five patients were survivors and 26 were nonsurvivors. Detailed baseline characteristics of the study population, stratified as survivors or nonsurvivors, are presented in Table 1. Microbiological identification of VAP is presented in Table 2.

**Table 1:** Baseline characteristics of 71 patients who developed ventilator-associated pneumonia

Parameter	Survivors (*n *= 45)	Nonsurvivors (*n *= 26)	Total (*n *= 71)	*P *value
Age (years)	58 ± 14	64 ± 16	60 ± 15	0.12
Acute Physiology and Chronic Health Evaluation II score	18 ± 6	22 ± 9	19 ± 7	0.06
Albumin level (mg/dl)	2.8 ± 0.6	2.4 ± 0.5	2.7 ± 0.6	0.01
Gender (%)				0.09
Male	66.7	46.2	59.2	
Female	33.3	53.8	40.8	
Origin (%)				0.25
Medical	51.1	65.4	56.3	
Surgical	48.9	34.6	43.7	
Onset (%)^a^				0.93
Early onset	22.2	23.1	22.5	
Late onset	77.8	76.9	77.5	
Chronic obstructive pulmonary disease (%)	17.7	26.9	19.7	0.59
Congestive heart failure (%)	17.8	26.9	21.1	0.37
Malignancy (%)	13.3	15.4	14.1	0.81
Histamine type-2 receptor antagonist (%)	66.7	57.7	63.4	0.45
Proton pump inhibitor (%)	22.2	34.6	26.8	0.26
Corticosteroids (%)	13.3	19.2	15.5	0.51
Dialysis (%)	11.1	19.2	14.1	0.35
Smoker (%)	37.8	38.5	38.0	0.95
Septic status (%)				0.01
Sepsis	66.7	15.4	47.9	
Severe sepsis	28.9	30.8	29.6	
Septic shock	4.4	53,8	22.5	

Data presented as the mean ± standard deviation or the percentage. ^a^Early onset is defined as occurring during the first 4 days of mechanical ventilation, and late onset as occurring 5 days or more after mechanical ventilation.

**Table 2:** Microbiological identification in 71 ventilator-associated pneumonia patients and mortality^a^

Microorganism	Survivors (*n *= 56^b^)	Nonsurvivors (*n *= 31^b^)	Total (*n *= 87^b^)
*Pseudomonas aeruginosa*	9 (16.1)	6 (19.4)	15 (17.2)
*Staphylococcus aureus *oxacillin resistant	8 (14.3)	5 (16.1)	13 (14.9)
*Staphylococcus aureus *oxacillin sensitive	7 (12.5)	1 (3.2)	8 (9.2)
*Stenotrophomonas maltophilia*	3 (5.4)	3 (9.7)	6 (6.9)
*Acinetobacter *sp	4 (7.1)	1 (3.2)	5 (5.7)
*Klebsiella pneumoniae*	2 (3.6)	3 (9.7)	5 (5.7)
*Enterobacter *sp	4 (7.1)	0	4 (4.6)
*Haemophilus *sp	4 (7.1)	0	4 (4.6)
*Escherichia coli*	0	2 (6.5)	2 (2.3)
*Citrobacter koseri*	2 (3.6)	0	2 (2.3)
*Proteus mirabilis*	2 (3.6)	0	2 (2.3)
Other	5 (8.9)	1 (3.2)	6 (6.9)
Nonidentified	6 (10.7)	9 (29.0)	15 (17.2)

Data presented as the frequency (%). Not all percentages add up to 100 because of rounding. ^a^Positive quantitative endotracheal aspirate when ≥10^5 ^colony-forming units/ml. ^b^We identified more than one microorganism in 11 patients who survived and in five patients who died. In total, 16 patients had more than one microorganism identified.

Eight patients were not included in the D4 analysis because six patients died before D4, one patient left the ICU before D4 and the copeptin measurement for one patient was not performed because a serum sample was not available.

**Figure 1:**
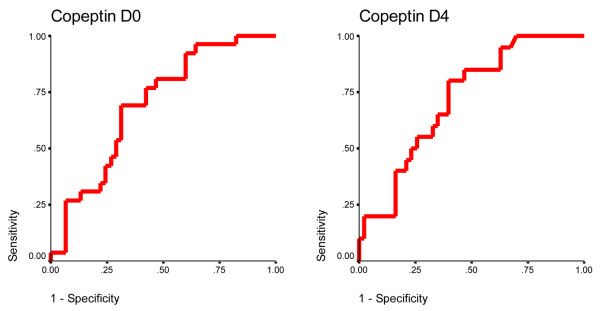
Receiver operating characteristic analysis of copeptin with respect to mortality prediction in ventilator-associated pneumonia patients. Data on the day of diagnosis of ventilator-associated pneumonia (D0) and on day 4 (D4) are shown.

Accuracy of copeptin to predict mortality in VAP patients on D0 and D4 was assessed by receiver operating characteristic curve analysis, as shown in Figure 1. The data are presented in Table 3. Copeptin had the slightly higher accuracy on D4 compared with D0. The area under the curve for Copeptin on D0 was 0.70 (standard deviation, 0.06; *P *= 0.006). For a threshold of 64.8 pmol/l (minimal false negative and false positive results), the sensitivity was 0.69 and the specificity was 0.69. The area under the curve for copeptin on D4 was 0.72 (standard deviation, 0.07; *P *= 0.006). Using a cutoff level of 43.0 pmol/l, the sensitivity was 0.80 and the specificity was 0.60.

**Table 3:** Prediction of mortality in patients with ventilator-associated pneumonia (*n *= 71): area under the curve of receiver operating curve characteristic plot analysis

Variable	Threshold (pmol/l)^a^	Sensitivity	Specificity	Area under the curve	Standard error	Asymptotic significance
Copeptin on day 0	64.8	0.69	0.69	0.70	0.06	0.006
Copeptin on day 4	43.0	0.80	0.60	0.72	0.07	0.006

^a^Optimal cutoff value (minimal false negative and false positive results).

**Figure 2:**
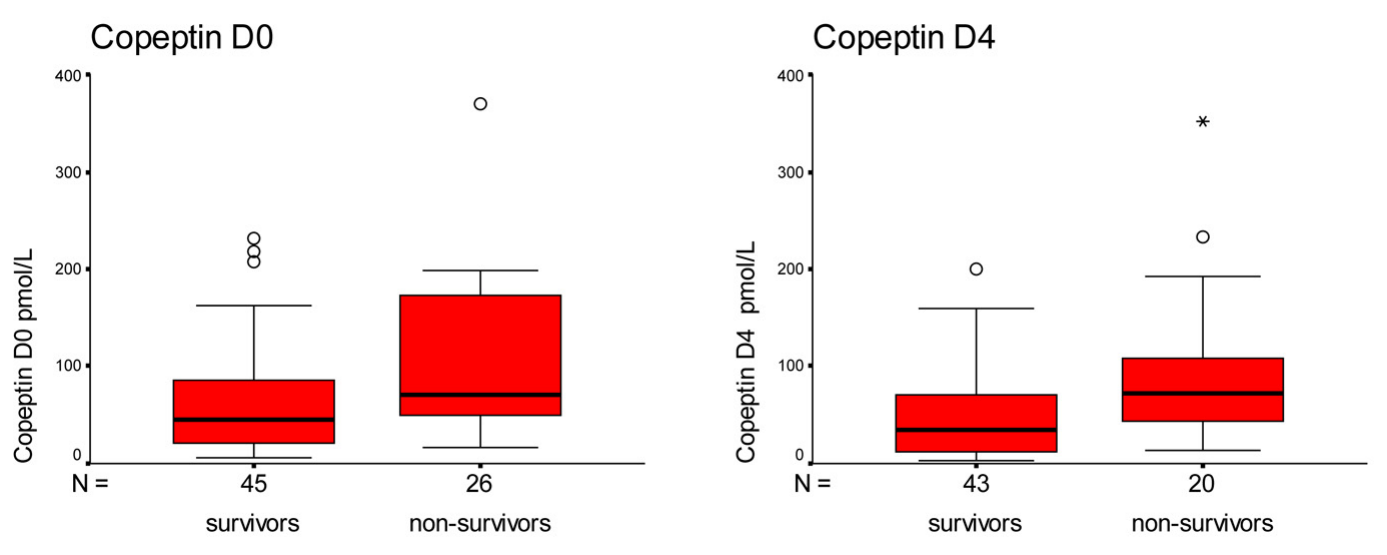
Copeptin levels in survivors and nonsurvivors. Box plots showing copeptin levels in survivors and nonsurvivors on day 0 (D0) and on day 4 (D4). Boxes represent the 25th to 75th percentiles. Circles and asterisks represent outliers.

Copeptin levels were lower in survivors compared with nonsurvivors on D0 (44.7 pmol/l and 74.2 pmol/l, respectively; *P *= 0.006). Similar results were found on D4 (34.5 pmol/l and 72.3 pmol/l, respectively; *P *= 0.006), as shown in Figure 2 and Table 4.

**Table 4:** Comparison of copeptin levels between survivors and nonsurvivors (Mann–Whitney test)

Variable		Median (pmol/l)	Interquartile range	*P *value
Copeptin on day 0	Survivor	44.7	7.8 to 81.6	0.006
	Nonsurvivor	74.2	12.3 to 136.1	
Copeptin on day 4	Survivor	34.5	2.6 to 66.4	0.006
	Nonsurvivor	72.3	38.6 to 106.0	

**Figure 3:**
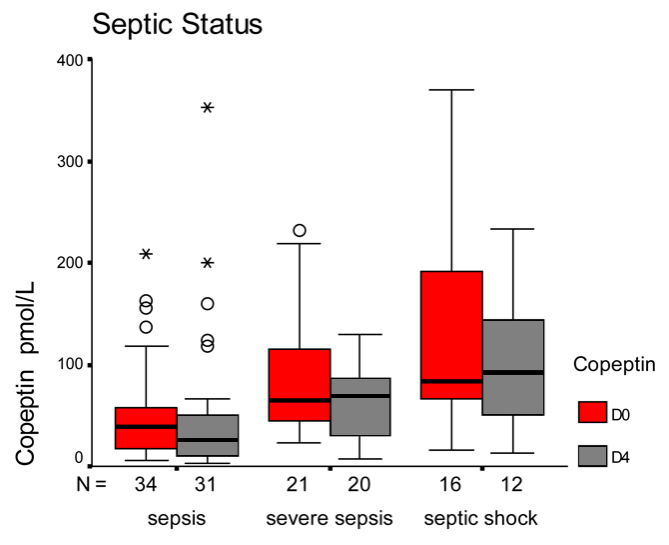
Copeptin levels in septic patients, severe sepsis patients, and septic shock patients. Box plots showing copeptin levels in septic patients, patients with severe sepsis and patients with septic shock on day 0 (D0) and on day 4 (D4). Boxes represent the 25th to 75th percentiles. Circles and asterisks represent outliers.

The influence of septic status on copeptin levels is shown in Table 5 and Figure 3. Values were higher in the septic shock group both for D0 and D4. Copeptin levels increased from sepsis to severe sepsis to septic shock both on D0 (41.2 pmol/l, 64.8 pmol/l, 84.2 pmol/l, respectively; *P *= 0.001) and on D4 (25.3 pmol/l, 68.7 pmol/l, 91.8 pmol/l, respectively; *P *= 0.009).

**Table 5:** Comparison of copeptin levels between different septic statuses (Kruskal–Wallis test)

Variable		Median (pmol/l)	Interquartile range	*P *value
Copeptin on day 0	Sepsis	41.2	17.2 to 65.9	0.001
	Severe sepsis	64.8	23.7 to 105.9	
	Septic shock	84.2	15.6 to 152.8	
Copeptin on day 4	Sepsis	25.3	2.1 to 48.5	0.009
	Severe sepsis	68.7	39.3 to 98.1	
	Septic shock	91.8	35.9 to 144.7	

Logistic regression analysis was used to determine the relation of risk factors to mortality. The variables included in the univariable logistic regression analysis for mortality were age, gender, APACHE II score, ln copeptin on D0 and ln copeptin on D4. In univariable analysis, ln copeptin on D0 (odds ratio, 2.32) and ln copeptin on D4 (odds ratio, 2.31) were predictors of mortality. There was a trend to significance for age, gender and APACHE II score.

The multivariable logistic regression model for mortality included the variables from the univariable analysis. The only variables that remained as independent predictors of death were ln copeptin D0 with an odds ratio of 1.97 (95% confidence interval, 1.06 to 3.69; *P *= 0.03), and ln copeptin D4 with an odds ratio of 2.39 (95% confidence interval, 1.24 to 4.62; *P *= 0.01) (Tables 6 and 7).

**Table 6:** Odds ratios for mortality in 71 ventilator-associated pneumonia patients on day 0: univariable and multivariable logistic regression analysis

Parameter	Univariable analysis		Multivariable analysis	
	
	Odds ratio (95% confidence interval)	*P *value	Odds ratio (95% confidence interval)	*P *value
Age	1.03 (0.99 to 1.06)	0.12	1.01 (0.98 to 1.05)	0.55
Gender, female	2.33 (0.87 to 6.28)	0.09	1.80 (0.62 to 5.23)	0.28
APACHE II score	1.07 (1.00 to 1.15)	0.06	1.05 (0.97 to 1.13)	0.23
ln copeptin on day 0	2.32 (1.25 to 4.29)	0.008	1.97 (1.06 to 3.69)	0.03

**Table 7:** Odds ratios for mortality in 71 ventilator-associated pneumonia patients on day 4: univariable and multivariable logistic regression analysis

Parameter	Univariable analysis		Multivariable analysis	
	
	Odds ratio (95% confidence interval)	*P *value	Odds ratio (95% confidence interval)	*P *value
Age	1.03 (0.99 to 1.06)	0.12	1.00 (0.97 to 1.05)	0.84
Gender, female	2.33 (0.87 to 6.28)	0.09	2.52 (0.75 to 8.45)	0.13
APACHE II score	1.07 (1.00 to 1.15)	0.06	1.07 (0.98 to 1.18)	0.14
ln copeptin on day 4	2.31 (1.25 to 4.25)	0.007	2,39 (1.24 to 4.62)	0.01

## Discussion

The current study demonstrates that copeptin levels are significantly higher in nonsurviving VAP patients compared with survivors. In multivariate logistic regression models of predictors of death, including age, sex, APACHE II score and copeptin level on the day of diagnosis of VAP (D0) and on day 4 (D4), copeptin was the only parameter that remained an independent predictor.

The role of neuroendocrine regulation in sepsis is under investigation. The hypothalamus is the integrating center for stress responses, and corticotropin-releasing hormone and vasopressin neurones convert stress signals to hormonal outputs [1]. Vasopressin is released into the portal circulation with corticotropin-releasing hormone in response to stress, and potentiates corticotropin-releasing hormone-induced ACTH secretion; thus, vasopressin and corticotropin-releasing hormone are both endogenous releasing peptides for ACTH [18]. Why we need both peptides for the regulation of glucocorticoid secretion, however, is not clear.

VAP and sepsis impose stress on patients, potentially promoting cardiovascular instability and an elevated demand for vasopressin and glucocorticoid secretion. Our data showed that copeptin levels increased progressively with the severity of sepsis. The levels appear to be consistent with findings reported elsewhere about copeptin levels in septic patients [4-6,19,20]. Septic shock VAP patients presented the highest values of copeptin and highest mortality in our sample. Septic shock and copeptin were colinear variables, and competed in the multivariate analysis, since they express the same phenomenon, as clinical and laboratorial variables, respectively. Since only 22.5% of our patients presented septic shock on D0, elevated copeptin levels at the diagnosis of VAP in a patient without septic shock could be helpful in prognostic assessment.

Copeptin might be used as a surrogate marker of the stimulated neuroendocrine regulation in septic patients.

Relatively low vasopressin plasma concentrations are suspected to contribute to cardiovascular failure in vasodilator shock [21-23], and copeptin levels are elevated in this condition. It was shown in a previous study that the AVP plasma concentration is lower in sepsis than the corresponding copeptin values [19]. As both peptides are initially secreted in an equimolar ratio, this could be an indication that AVP is rapidly consumed in extreme physiological conditions, thus resulting in a relative AVP insufficiency. It is possible that AVP therapy could be useful particularly in those patients who have this discrepant copeptin/AVP ratio. High copeptin in the presence of vasodilatory shock may indicate insufficient endogenous AVP production and may warrant exogenous substitution. This hypothesis needs to be addressed in a future prospective study, however, as we did not measure AVP in the present study.

Further studies must evaluate whether implementation of exogenous vasopressin therapy in patients with vasodilatory shock should also be guided by endocrinologic investigation or exclusively by cardiovascular investigations.

In the present study we demonstrated copeptin is an independent predictor of mortality in VAP. In such a condition it is essential to assess the disease severity to optimize clinical decision-making and therapy.

Our data should be interpreted in light of certain limitations. First, copeptin comes from the same precursor as mature AVP, which is already well associated with hemodynamic changes and patient outcome. The measurement of mature AVP, however, is subject to considerable challenges, and has therefore not reached clinical routine in the context of rapid measurements in the ICU setting. Here the stability and longer *ex vivo *half-life of copeptin is a practical advantage, which makes it easier to determine in the clinical laboratory. Second, our sample size is not large enough for a stronger analysis, and adrenal failure was therefore not assessed. Third, our study was not designed to include a control group, which limits positive and negative predictive value assessment. Finally, the use of crude mortality instead of attributable mortality is another limitation, but it also avoids variability and confounding interpretation.

Prior to the present study no published information existed about the behavior of copeptin in patients with VAP. Copeptin may represent a novel tool to assess prognosis in VAP. Additional studies are warranted to investigate these findings and to further define the potential impact of strategies based on biomarkers in improving VAP outcomes.

## Conclusion

Copeptin levels increase progressively with the severity of sepsis in VAP patients and are independent predictors of mortality in this condition.

## Key messages

• Copeptin levels increase progressively with the severity of sepsis and are independent predictors of mortality in VAP.

## Abbreviations

APACHE = Acute Physiology and Chronic Health Evaluation; AVP = arginine vasopressin; CPIS = Clinical Pulmonary Infection Score; ICU = intensive care unit; QEA = quantitative endotracheal aspirate; VAP = ventilator-associated pneumonia.

## Competing interests

NGM and JP are employees of BRAHMS AG (Hennigsdorf/Berlin, Germany), the manufacturer of the copeptin assay. MM has received remuneration for holding lectures on the topic of inflammation markers by BRAHMS AG, Germany. The authors declare that there are no further competing interests.

## Authors' contributions

RS developed the study design, coordinated its implementation and was responsible for patient recruitment as well as data collection. NGM and JP carried out laboratory tests. RS and PJZT carried out the statistical analysis. RS, NGM, JP, MM and PJZT participated in interpretation/discussion of results and drafted and revised the manuscript. All authors read and approved the final manuscript.
